# Associations of Intimacy, Partner Responsiveness, and Attachment-Related Emotional Needs With Sexual Desire

**DOI:** 10.3389/fpsyg.2021.665967

**Published:** 2021-06-21

**Authors:** Jacques J. D. M. van Lankveld, Marieke Dewitte, Peter Verboon, Susan A. H. van Hooren

**Affiliations:** ^1^Faculty of Psychology, Open University of the Netherlands, Heerlen, Netherlands; ^2^Clinical Psychological Science, Maastricht University, Maastricht, Netherlands

**Keywords:** sexual desire, attachment-related emotional needs, partner responsiveness, intimacy, anxious adult attachment, avoidant adult attachment

## Abstract

In this online cross-sectional survey study in a large community sample we investigated the associations between attachment-related relational needs, partner responsiveness, intimacy, and sexual desire, using structural equation modeling. Participants were heterosexual and non-heterosexual women and men. Intimacy and partner responsiveness correlated positively with sexual desire in all subsamples. Anxious attachment-related relational needs correlated positively with sexual desire. Avoidant attachment-related relational needs correlated negatively with sexual desire. Anxious and avoidant attachment-related needs, however, did not moderate the association between intimacy and sexual desire. Individuals with problems of low sexual desire may benefit from an emphasis in sex therapy on ways to increase (perceptions of) intimacy.

## Introduction

Sexual desire is a central aspect of sexual functioning (Mark and Lasslo, 2018). Lower than desirable level of sexual desire is a highly prevalent sexual problem in many countries (Štulhofer et al., 2005; Kedde, 2012; McCabe et al., 2016). The incentive motivation theory (Singer and Toates, 1987; Ågmo, 1999; Toates, 2014) postulates that sexual motivation, giving rise to the conscious experience of sexual desire, emerges upon perceiving external or internal erotic stimuli that are appraised as sexually meaningful and rewarding (Janssen et al., 2000). Sexual motivation is thus hypothesized to be crucially driven by the expectation of reward. An important reward is the emotional intimacy that romantic partners can experience during a sexual encounter. An influential definition of emotional intimacy is that of Sternberg (1986). It involves the experience of strong feelings of closeness, connectedness and bonding. Intimacy thus serves as an incentive for sexual motivation. In line with the incentive-motivation theory, the circular model of female sexual responding (Basson, 2000, 2003) claims that intimacy serves both as a trigger for sexual desire, and as a reward created by the experience of sexual arousal and - in particular - of orgasm (Basson, 2000). The circular model is also considered relevant for male sexual functioning (Štulhofer et al., 2013; Connaughton et al., 2016), but its relevance for women and men who do not experience sexual dysfunction has been questioned (Sand and Fisher, 2007; Giles and McCabe, 2009). Empirical evidence of the association of intimacy and sexual functioning is still scarce (McCabe, 1997; Birnbaum et al., 2007; Rubin and Campbell, 2012).

The appraisal of the reward value of emotional intimacy is crucially guided by one’s attachment orientation (Feeney and Noller, 2004; Mikulincer and Shaver, 2007; Birnbaum, 2010; Dewitte, 2012). Adult attachment theory postulates that securely attached individuals enjoy high levels of emotional intimacy and experience partnered sexual activity with self-confidence (Shaver and Mikulincer, 2012). They are able to be sexual for the mere joy of it. For them, partnered sexual activity is not necessary for satisfying attachment-based interpersonal needs. Anxiously attached individuals, however, may need sexual contact for securing proximity with their partner, based on their strong need to feel loved and protected. They may, nevertheless, have difficulties to enjoy sexual contact and may continue feeling worried and anxious related to sexual activity (Dewitte, 2012). Avoidantly attached individuals tend to avoid sexual activity with their romantic partner because they feel overwhelmed during high levels of emotional closeness. They prefer sexual contact in a more emotionally detached way or with a sexual partner outside of a romantic relationship (Brennan and Shaver, 1995). Such preferences of insecurely attached individuals serve to protect their sense of control over the interaction and help them to feel autonomous (Mikulincer and Shaver, 2007; Mikulincer et al., 2010; Dewitte, 2012).

Closely related to the concepts of intimacy and attachment is the notion of perceived partner responsiveness. Perceived partner responsiveness is defined as the extent to which one experiences the partner as being responsive to one’s emotional needs (Reis, 2013). It plays an important role in shaping intimate interactions between partners. The perceptions of partner availability and responsiveness play a pivotal role in adult attachment theory (Mikulincer et al., 2010; Dewitte, 2012). When distress and perceived threat levels increase in insecurely attached adults, the attachment system is activated and the proximity of an attachment figure is pursued. When this figure is perceived as available and responsive, security feelings increase, and the activation of the attachment system ceases. In case the attachment figure is perceived as unavailable, which is characteristic of individuals with anxious or avoidant attachment orientations (Segal and Fraley, 2016), distress levels remain high, the feelings of insecurity persist, and alternative strategies are invoked to deal with insecurity: hyperactivating strategies in anxiously attached people and deactivating strategies in avoidantly attached people.

**FIGURE 1:**
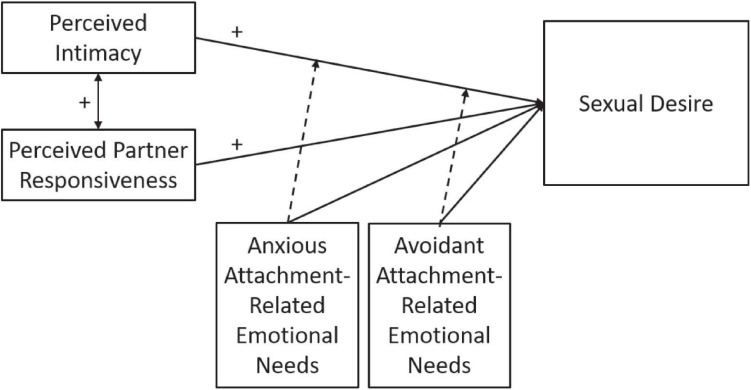
A conceptual model of the determinants of sexual desire.

Perceived intimacy and responsiveness of the partner are thus assumed to vary as a function of one’s attachment-related needs, and to serve as incentives for sexual motivation, which is consciously experienced as sexual desire. Given that attachment orientation determines the reward value of intimacy and partner responsiveness, it may play an important role in triggering and regulating sexual desire. In long-term relationships reduced intimacy and partner responsiveness may be risk factors for the development of problems regarding sexual desire and satisfaction. Enhancing the understanding of the associations of intimacy, partner responsiveness, attachment orientation and sexual desire is required to enable the creation of preventive interventions to maintain satisfying sexuality in long-term relationships. Figure 1 visualizes the associations postulated in the conceptual model that will be tested in the current study.

These associations between intimacy, attachment and sexual desire have been investigated in a longitudinal study in which newly-wed couples were followed over a period of 8 months (Mizrahi et al., 2016). When male partners displayed sexual desire in a videotaped interaction with their female spouse at the onset of the study, this predicted later decline in attachment-related relationship insecurities, both of themselves and of their partner. In contrast, displays of sexual desire by female partners inhibited the later decrease of their male partner’s attachment-related relationship insecurities, whereas displays of intimacy of female partners predicted later decline in their partner’s attachment-related relationship insecurities. Other empirical tests of the association between intimacy, attachment and sexual desire, specifically considering perceived partner responsiveness as important determinant of both attachment and sexual desire, are thus far lacking.

The bivariate relationships between different elements of the model were investigated in several studies. In a longitudinal daily diary study, intimacy was found to increase the odds of partnered sexual activity in both men and women (Dewitte et al., 2015). Sexual desire was also found to mediate the link between intimacy and sexual satisfaction (Muise et al., 2013). In an ecological momentary assessment study among non-clinical women and men in long-term relationships, the association between perceived intimacy and partnered sexual activity was fully mediated by sexual desire (van Lankveld et al., 2018). These effects were similar in both genders. The direct effect of intimacy on sexual partner interaction was not significant. Another study using ecological momentary assessment methodology (Shrier and Blood, 2016), however, found gender differences in the associations between intimacy and sexual desire in emerging heterosexual couples. In male partners, higher relationship quality and higher enjoyment of physical intimacy were associated with their own higher and more stable sexual desire and with the stability of reported emotional intimacy of their female partners. In female partners, in contrast, the associations between momentary sexual desire and the relationship outcomes were not significant. Ferreira et al. (2014) also found gender differences in the associations of emotional intimacy and sexual desire with relationship satisfaction in cross-sectional data.

Regarding the association between partner responsiveness and attachment, it has been argued that empathic skills and the responsiveness of one partner to the attachment-related needs of the other partner may buffer the couple’s affect-regulating strategies (Ebesu Hubbard, 2001), promote non-defensive reactions to failure or conflict (Caprariello and Reis, 2011), and increase intimacy between partners (Laurenceau et al., 1998; Manne et al., 2004). This hypothesis was supported in research showing that partner responsiveness played a significant role in attachment-related interactions in a 1-year longitudinal study of relationship dynamics (Segal and Fraley, 2016). Compared with securely attached individuals, insecurely attached individuals were less likely to experience their partner as responsive. Relevant to the present study, partner responsiveness was found to increase sexual desire, particularly in women (Birnbaum et al., 2016). In a study among randomly paired strangers (Birnbaum and Reis, 2012), participants who perceived their partner as more responsive reported higher interest in sex with this partner. This effect was most prominent in participants with low avoidant attachment.

Although several bivariate relationships between elements of the model presented above were empirically investigated, a comprehensive test of the full model, however, has – to the best of our knowledge – not been conducted thus far. Furthermore, research into the associations of intimacy and attachment orientation with sexual desire in non-heterosexual samples is scarce. In this field of interest, many investigations have sampled heterosexual individuals, and emergent heterosexual couples in student populations, whereas larger community studies are lacking, potentially producing sampling bias.

### The Present Study

In the present study we aimed to investigate the associations between intimacy, partner responsiveness, and attachment orientation on the one hand, and sexual desire on the other hand (see Figure 1). We focused specifically on dyadic sexual desire (the desire to be sexual with one’s partner; as opposed to the solitary desire to be sexual with oneself) in individuals with a committed romantic relationship, given the interpersonal nature of our predictor variables. We also aimed to investigate the moderation of the association between intimacy and sexual desire by anxious and avoidant attachment. As discussed in the introduction, we assume that real-time experienced attachment-related emotional needs can be used as a proxy for attachment orientation. The present study was performed among partnered, heterosexual and non-heterosexual women and men in a larger community sample, using online survey methodology. Specifically, we tested the following hypotheses: (1) Intimacy is positively associated with sexual desire; (2) Partner responsiveness is positively associated with sexual desire; (3) Intimacy and partner responsiveness are positively associated; (4) Anxious attachment is positively associated with sexual desire; (5) Avoidant attachment is negatively associated with sexual desire; and (6) The associations of intimacy and partner responsiveness with sexual desire are moderated by attachment orientation, with higher anxious attachment showing stronger associations and higher avoidant attachment showing weaker associations. In explorative analyses, these associations were investigated in subsamples defined by gender and sexual orientation. The effects of age, duration of relationship, and education on sexual desire were examined in a preliminary analysis and added to the statistical model when found to explain significant portions of the variance.

## Materials and Methods

### Participants

Participants in our community-based sample were visitors of a popular Dutch website https://www.quest.nl, who completed a survey, https://www.quest.nl/test/hoe-intiem-is-jouw-liefde. The website offers a large number of brief surveys on a variety of topics. For the analyses in the present study, data were selected of participants meeting the inclusion criteria of 18 years and older and reporting being in a romantic partner relationship.

### Procedure

The study was promoted in various target groups to stimulate participating in the survey. Notifications were posted on the Quest survey website itself, inviting interested visitors to the current survey. It was also promoted in a digital newsletter of Quest Psychology, a popular monthly magazine for the general public, and posted on corporate social media (Facebook and Twitter) three times with 3 weeks in between. It was also promoted by the Open University on corporate social media (Facebook, Instagram, LinkedIn, and Twitter) four times with 2 weeks in between, with paid advertisements on Facebook, LinkedIn in corporate and psychology groups during 2 months, in newsletters and in follow-up mailings to persons requesting to send brochures for courses, and on the corporate intranet system. The study protocol and procedures were approved by the Ethical Review Board of the Open University.

Participants completed the survey via the website of https://www.quest.nl/test. The survey started with a brief introductory text, containing a link to a general document, stating that the participant’s data would be used for scientific enquiry. The names and affiliations of the researchers involved in the survey were disclosed. Informed consent was provided by clicking on the “Start test” button. Each participant then first selected the version of the questionnaire that matched their relationship status, respectively, the version for participants with a romantic relationship or the version for singles. Singles’ data were not used for further analyses in this study. For each question a new page was presented containing the question and the answering options. After clicking on the desired option, this choice was confirmed by clicking on the “Next” button.

The questionnaire started with four questions that were not part of the study, but were added as “appetizers,” for example “How strongly do you endorse the following statement: I share all my feelings with my partner?” These questions were included to familiarize participants with the topic of intimacy in one’s relationship. Next the survey questions were presented. Immediately following the answer to the last question, a debriefing statement was presented on screen that indicated whether the given responses placed the participant at the sample’s low, middle, or high end of the intimacy scale. No momentary compensation was given.

### Instruments

Demographic questions assessed participant’s gender, sexual orientation, age, relationship duration, and level of education. Gender identity was assessed as ‘male,’ ‘female,’ and ‘other/not disclosed.’ Note that with this question it is not possible to rule out that the respondent is transgender. Participants were asked to report their partner’s gender. The reply to this question was used to derive the participant’s sexual orientation. Bisexual orientation could therefore not be distinguished from heterosexual and gay/lesbian orientation. Other survey items aimed to assess aspects of intimacy, partner responsiveness, anxious and avoidant attachment-related emotional needs, and sexual desire. Due to the protocol of the Quest survey environment, the total number of possible questions was limited. All items took the form of statements. Participants indicated their level of endorsement of these statements on a 7-point Likert-type scale with response categories ranging from 1 (“definitely not”) to 7 (“yes, definitely”). Within the constraints of the Quest survey protocol, it was not possible to assess the key concepts using validated measures of sexual desire, intimacy, partner responsiveness, and attachment orientation. Instead, each concept was assessed using a limited number of items, taken from validated instruments if possible.

#### Sexual Desire

Sexual desire was assessed using two items, formulated as “At this moment I would like to have sex with my partner” and “At this moment I am open for my partner’s sexual initiative” (see Table 1). The first item was adapted from the Sexual Desire Inventory (Spector et al., 1996); the second was adapted from the Sexual Interest and Desire Inventory–Female (SIDI-F; Clayton et al., 2006). Items wordings refer to both the proactive and the responsive dimensions of sexual desire. As the items were responded to in the context of an individually completed survey without the partner playing an active role, they are both considered to reflect spontaneous sexual desire. A sexual desire score was calculated by summarizing the scores on the two items. The wording of the items, instructing the participants to rate their momentaneously experienced level of sexual desire, was chosen to focus the participant’s attention on experiences in the present moment and thus avoid potential memory bias that is associated with retrospectively rating one’s experiences over prolonged periods of time (Bolger et al., 2003). The internal consistency of the sexual desire scale was satisfactory in the full sample (Cronbach’s α = 0.81), and αs ranging from 0.74 to 0.81 in the four subsamples defined by gender and sexual orientation.

**TABLE 1:** Survey item wordings.


**Construct**	**Dutch wording**	**English translation**
Sexual desire	Op dit moment heb ik zin in seks met mijn partner Op dit moment sta ik open voor seksueel initiatief van mijn partner	At this moment I would like to have sex with my partner At this moment I am open for my partner’s sexual initiative
Perceived intimacy with partner	Als ik aan mijn partner denk voel ik emotionele afstand Als ik aan mijn partner denk voel ik liefde Als ik aan mijn partner denk voel ik warmte Als ik aan mijn partner denk voel ik verbondenheid	I feel distance when I think of my partner I feel love when I think of my partner I feel warmth when I think of my partner I feel connected when I think of my partner
Perceived partner responsiveness	My partner now accepts me as I am My partner now gives me the emotional support I need My partner is now very sensitive to my emotional needs	Op dit moment waardeert mijn partner mij zoals ik ben Op dit moment krijg ik van mijn partner de emotionele steun die ik nodig heb Op dit moment voelt mijn partner mij goed aan
Attachment-related needs	Ik vind het erg als mijn partner mij kwetst Ik heb graag dat mijn partner mij emotioneel steunt Ik heb graag dat mijn partner aanvoelt wat ik nodig heb Ik ben graag onafhankelijk van mijn partner	I am afraid of being emotionally hurt by my partner I want my partner to support me emotionally I want my partner to be sensitive to my emotional needs I want to feel independent of my partner

#### Perceived Intimacy

Perceived intimacy was assessed using four survey items (see Table 1). The items were selected from a set used previously in our lab (van Lankveld et al., 2018) in a longitudinal study on intimacy and sexuality. The item wordings follow Sternberg (1986) definition of intimacy as the experience of strong feelings of closeness, connectedness, and bondedness. Reliability was satisfactory, with Cronbach’s α = 0.79 in the full sample, and αs ranging from 0.75 to 0.79 in the four subsamples defined by gender and sexual orientation. A sum score of the constituent four items was calculated, representing Perceived Intimacy with the partner.

#### Perceived Partner Responsiveness

Perceived partner responsiveness was assessed using three items (see Table 1). The item wordings were adapted from selected items of the Perceived Partner Responsiveness Scale (PPRS; Reis et al., 2011, 2018). Reliability was satisfactory, with Cronbach’s α = 0.84 in the full sample, and αs ranging from 0.82 to 0.86 in the four subsamples defined by gender and sexual orientation. A sum score was calculated to represent Perceived Partner Responsiveness.

#### Attachment-Related Relational Needs

Respondent’s relational needs, related to anxious and avoidant attachment, were assessed using four items (see Table 1). The items were adapted from selected items from the Experiences in Close Relationships (ECR; Brennan et al., 1998; Conradi et al., 2006), and the Need Satisfaction scale of La Guardia et al. (2000). They were formulated as needs with regard to the partners relational behavior to reflect the core beliefs underlying anxious (“I want my partner to support me emotionally”) and avoidant (“I want to feel independent of my partner”) attachment orientations. PCA with varimax rotation was conducted in the full sample. The solution that best represented the data comprised two components with eigenvalues >1.0, together explaining 61.7% of the variance. The first factor was termed “Anxious Attachment-Related Needs” (eigenvalue = 1.348; proportion of explained variance = 33.7%) and received loadings from two items (“I want my partner to be sensitive to my emotional needs” (rotated component loading = 0.802), and “I want my partner to support me emotionally” (rotated component loading = 0.791). The second factor was termed “Avoidant Attachment-Related Needs” (eigenvalue = 1.120; proportion of explained variance = 28.0%). It received loadings from the other two items (“I am afraid of being emotionally hurt by my partner” (rotated component loading = 0.765), and “I want to feel independent of my partner” (rotated component loading = 0.715). Similar factor structures were found in repeated PCAs in the subsamples defined by gender and sexual orientation. A sum score of the constituent items was calculated to represent Anxious Attachment-Related Needs and Avoidant Attachment-Related Needs.

### Statistical Analysis

Descriptive data of participants’ demographic characteristics were analyzed across gender and sexual orientation subgroups. Preliminary analyses were conducted, using multiple regression, to examine the associations of age, relationship duration, and level of education, and subsequently of gender and sexual orientation, with sexual desire. In case significant associations were found, age and/or relationship length were entered as covariates in further analyses, to examine their contribution. Analyses were performed in the full sample, and additionally in subsamples of heterosexual and non-heterosexual men and women. Bivariate correlations between all study variables were calculated, both for hypothesis testing and the evaluation of possible bivariate collinearity.

The main study hypotheses predicting (moderation of) bivariate associations were tested using structural equation modeling, using Lavaan (Rosseel, 2012) in R (R_Core_Team, 2016). For these analyses, the sample was randomly split in halves. One sample was used to optimize the fit of the theoretical model, while the second was used to evaluate the robustness of the optimized model by testing for measurement invariance across the two samples. The regression coefficients of the predictors in the solutions in the two subsamples and the explained variance of proactive and responsive sexual desire were presented. A bootstrap analysis was done on the validation set to obtain 95% confidence intervals. The relative chi-square (χ^2^/*df*), the Tucker Lewis Index (TLI), the Standardized Root Mean Square Residual (SRMR), and the root-mean-square error of approximation (RMSEA) were used as fit indices. Cut-off values of <2 for the relative chi-square (Arbuckle, 2011), >0.95 for the TLI (Tabachnick and Fidell, 2007; Kline, 2011) and <0.06 for the RMSEA and the SRMR (Arbuckle, 2011) indicate a good fit, and an adequate fit is indicated when TLI exceeds 0.90, and when RMSEA and SRMR are below 0.08 (Tabachnick and Fidell, 2007; Arbuckle, 2011; Kline, 2011). Effect sizes were evaluated against Cohen’s (1988) criteria.

## Results

### Preliminary Results

Participants who met the inclusion criteria (*N* = 10202) were 7701 women (Mean age = 29.7, SD = 9.5; range = 18–75) and 2501 men (Mean age = 33.3, SD = 11.9; range = 18–76). Mean relationship duration was 6.9 years in women (SD = 7.8 years), and 8.7 years (SD = 10.0 years) in men. Demographic characteristics are shown in Table 2. Note that the female subsample size here is smaller (*N* = 7692) due to missing demographic data of nine participants. Male participants were older than female participants [*t*(3594) = 13.6, *p* < 0.001], and reported longer relationship duration [*t*(2642) = 7.1, *p* < 0.001]. Heterosexual participants reported longer relationship duration than non-heterosexual participants [*t*(1058) = 2.1, *p* = 0.033]; there were no significant age differences in both groups. Education levels did not differ across gender and sexual orientation.

**TABLE 2:** Participants’ demographic characteristics.

	**Women**			**Men**		
	**Heterosexual (*N* = 6934; 90.2%) *M (SD)***	**Non-heterosexual (*N* = 758; 9.8%) *M (SD)***	**Total (*N* = 7692) *M (SD)***	**Heterosexual (*N* = 2134; 85.3%) *M (SD)***	**Non-heterosexual (*N* = 367; 14.7%) *M (SD)***	**Total (*N* = 2501) *M (SD)***
Age	29.7 (9.5)	29.8 (9.8)	29.7 (9.5)	33.4 (12.1)	32.3 (11.3)	33.3 (11.9)
Relationship duration (y)	7.0 (7.9)	6.3 (7.4)	6.9 (7.8)	8.9 (10.1)	7.8 (9.2)	8.7 (10.0)
Level of education Lower secondary Higher secondary Professional College/University Other	12.4% 24.5% 38.2% 21.8% 3.2%	11.7% 25.6% 39.3% 20.7% 2.6%	12.3% 24.6% 38.3% 21.7% 3.2%	12.0% 26.3% 39.0% 19.7% 3.0%	9.6% 22.1% 41.4% 24.3% 2.7%	11.7% 25.7% 39.4% 20.4% 2.9%

Table 3 displays the mean scores for sexual desire and the other variables of interest. A stepwise multiple linear regression analyses was conducted with sexual desire as dependent variable, and participants’ age, relationship duration and level of education as predictor variables. Level of education was treated as a quasi-continuous variable. The regression model predicting level of sexual desire was significantly different from a model containing only a constant [*F*(3,7116) = 77.7, *p* < 0.001], but the effect size was small (*R*^2^ = 0.032). Age contributed significantly and positively to the prediction of level of sexual desire (β = 0.064, *t* = 4.2, *p* < 0.001), while relationship duration made a significant negative contribution (β = −0.212, *t* = −14.0, *p* < 0.001). Higher age and shorter relationship predicted higher level of sexual desire. Level of education was not a significant predictor of sexual desire.

**TABLE 3:** Participants’ scores of sexual desire, perceived intimacy, partner responsiveness, and attachment-related needs.

	**Women**			**Men**		
	**Heterosexual (*N* = 6943; 90.2%) *M (SD)***	**Non-heterosexual (*N* = 758; 9.8%) *M (SD)***	**Total (*N* = 7701) *M (SD)***	**Heterosexual (*N* = 2134; 85.3%) *M (SD)***	**Non-heterosexual (*N* = 367; 14.7%) *M (SD)***	**Total (*N* = 2501) *M (SD)***
Sexual desire^*a*^	9.7 (3.6)	9.8 (3.5)	9.7 (3.6)	11.4 (3.0)	10.9 (3.1)	11.4 (3.0)
Perceived intimacy^*b*^	25.4 (4.2)	25.5 (4.0)	25.4 (4.2)	25.2 (4.2)	25.2 (4.0)	25.2 (4.2)
Perceived partner responsiveness^*c*^	17.4 (3.6)	17.7 (3.4)	17.4 (3.6)	17.4 (3.7)	17.2 (3.7)	17.4 (3.7)
Anxious attachment-related needs^*d*^	12.5 (1.7)	12.4 (1.7)	12.5 (1.7)	12.0 (2.0)	12.2 (1.7)	12.0 (1.9)
Avoidant attachment-related needs^*d*^	8.0 (2.7)	8.0 (2.8)	8.0 (2.7)	7.2 (2.7)	7.6 (2.6)	7.3 (2.7)

*^*a*^Scoring range: 2–14; ^*b*^Scoring range: 4–28; ^*c*^Scoring range: 3–21; ^*d*^Scoring range: 2–14.*

### Gender and Sexual Orientation Differences in Sexual Desire

To investigate differences in levels of sexual desire with regard to gender and sexual orientation a 2 × 2 analysis of covariance was performed with sexual desire as dependent variable, with gender and sexual orientation as within-group factors, and with age and relationship duration as covariates. Evaluation of the assumptions for analysis of covariance revealed satisfactory results. After adjustment for the covariates, sexual desire varied significantly by gender [*F*(1,7114) = 158.1, *p* < 0.001], and by sexual orientation [*F*(1,7114) = 5.4, *p* = 0.021], both with a small or extremely small effect size (respectively, $\eta_{\text{p}}^{2}$ = 0.022; $\eta_{\text{p}}^{2}$ = 0.001). The interaction effect of gender and sexual orientation was significant, with an extremely small effect size [*F*(1,7114) = 7.0, *p* = 0.008, $\eta_{\text{p}}^{2}$ = 0.001]. Compared to female participants, higher levels of sexual desire were reported by male participants. The effects on sexual desire of sexual orientation and the interaction of gender and sexual orientation were too small for meaningful interpretation.

### Associations of Perceived Intimacy, Partner Responsiveness, and Attachment-Related Emotional Needs With Sexual Desire

The results of bivariate correlation analysis in the full sample are shown in Table 4, upper panel. Perceived intimacy and perceived partner responsiveness correlated positively and significantly with sexual desire (both *r* = 0.25, *p* < 0.001), with a medium effect size. Perceived intimacy and partner responsiveness were strongly correlated (*r* = 0.73, *p* < 0.001). To examine whether partner responsiveness still contributed independently to the prediction of sexual desire, in addition to perceived intimacy, multiple linear regression analysis was performed in the random-split files, with sexual desire as to-be-predicted variable, with perceived intimacy entered in the first step, and the interaction term of perceived intimacy and partner responsiveness in the second step. In both data sets the interaction of intimacy and partner responsiveness significantly added explanatory value to the prediction of sexual desire by perceived intimacy alone [Data set 1: *R*^2^_*Change*_ = 0.01, *F*_*Change*_(1,5061) = 56.4, *p* < 0.001; Data set 2: *R*^2^_*Change*_ = 0.005, *F*_*Change*_(1,5135) = 26.0, *p* < 0.001].

**TABLE 4:** Bivariate correlations of sexual desire, perceived intimacy, perceived partner responsiveness, and attachment-related needs.

		**1**	**2**	**3**	**4**	**5**
*Full sample (N* = *10202)*						
1	Sexual desire	–				
2	Perceived intimacy	0.25^*c*^	–			
3	Perceived partner responsiveness	0.25^*c*^	0.73^*c*^	–		
4	Anxious attachment-related needs	0.07^*c*^	0.21^*c*^	0.19^*c*^	–	
5	Avoidant attachment-related needs	−0.05^ c^	−0.32^ c^	−0.35^ c^	−0.02	–
*Heterosexual women (N* = *6943)*						
1	Sexual desire	–				
2	Perceived intimacy	28^ c^	–			
3	Perceived partner responsiveness	0.27^ c^	0.73^ c^	–		
4	Anxious attachment-related needs	0.07^ c^	0.20^ c^	0.18^ c^	–	
5	Avoidant attachment-related needs	−0.03^*a*^	−0.34^ c^	−0.37^ c^	−0.03^*a*^	–
*Non-heterosexual women (N* = *758)*						
1	Sexual desire	–				
2	Perceived intimacy	0.28^ c^	–			
3	Perceived partner responsiveness	0.24^ c^	0.72^ c^	–		
4	Anxious attachment-related needs	0.15^ c^	0.17^ c^	0.14^ c^	–	
5	Avoidant attachment-related needs	−0.06	−0.33^ c^	−0.36^ c^	−0.01	–
*Heterosexual men (N* = *2134)*						
1	Sexual desire	–				
2	Perceived intimacy	0.20^ c^	–			
3	Perceived partner responsiveness	0.19^ c^	0.71^ c^	–		
4	Anxious attachment-related needs	0.17^ c^	0.26^ c^	0.26^ c^	–	
5	Avoidant attachment-related needs	−0.02	−0.29^ c^	−0.30^ c^	−0.04	–
*Non-heterosexual men (N* = *367)*						
1	Sexual desire	–				
2	Perceived intimacy	0.16^ c^	–			
3	Perceived partner responsiveness	0.22^ c^	0.75^ c^	–		
4	Anxious attachment-related needs	−0.01	0.17^ c^	0.12^*a*^	–	
5	Avoidant attachment-related needs	0.00	−0.24^ c^	−0.29^ c^	−0.05	–

*^*a*^*p* < 0.05, ^*b*^*p* < 0.01, ^*c*^*p* ≤ 0.001.*

Anxious attachment-related needs were positively, but weakly, correlated with sexual desire (*r* = 0.07, *p* < 0.001). Avoidant attachment-related needs showed a weak negative correlation with sexual desire (*r* = −0.05, *p* < 0.001). Anxious attachment-related needs were positively correlated with perceived intimacy (*r* = 0.21, *p* < 0.001) and perceived partner responsiveness (*r* = 0.19, *p* < 0.001). Avoidant attachment-related needs were negatively associated with perceived intimacy (*r* = −0.32, *p* < 0.001) and perceived partner responsiveness (*r* = −0.35, *p* < 0.001). Anxious and avoidant attachment were uncorrelated (*p* > 0.05). The correlation analysis was repeated in the four subgroups, see lower panels in Table 4. The pattern of associations was similar in all respects, except for the correlation between anxious attachment-related needs and sexual desire, that was positive and significant in the full sample and all subsamples, but not significant in the subsample of non-heterosexual men (*p* > 0.05).

### Moderation of the Association of Perceived Intimacy and Partner Responsiveness With Sexual Desire by Attachment-Related Relational Needs

The data were randomly split. Evaluation of the assumptions of analysis of covariance resulted in deletion of 245 multivariate outliers based on their Mahalanobis distance scores (*p* < 0.001). The test data set contained 5034 cases for hypothesis testing; the validation set contained 4923 cases.

**FIGURE 2:**
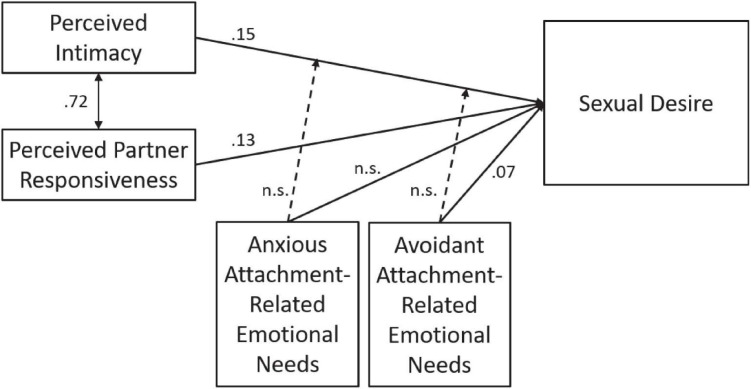
Final structural equation model. The parameter estimates for the regression coefficients are based on validation data. Anxious Attachment, Anxious Attachment Based Needs; Avoidant Attachment, Avoidant Attachment Based Needs.

The observed correlation between perceived intimacy and partner responsiveness had a large effect size (*r* = 0.72, *p* < 0.001). The interaction term between intimacy and partner responsiveness in the first test of the model was very small and therefore not further investigated as an independent moderator of the association of intimacy and sexual desire. Using the test dataset we added covariates and covariances between the predictors to improve the fit of the model. Based on the modification indices two covariates were added to the model: Gender and Relationship duration. Besides the covariance between these two covariates, also ten (out of fifteen) covariances between the predictors were added, which implies that five covariances were assumed to be zero. These five covariances were: between partner responsiveness and the interaction of intimacy × anxious attachment (1), between avoidant attachment and anxious attachment (2), between avoidant attachment and the interaction of intimacy × anxious attachment (3), between anxious attachment and the interaction of intimacy × avoidant attachment (4), and finally between the interaction of intimacy × avoidance attachment and the interaction of intimacy × anxious attachment (5). The final model is shown in Figure 2.

The model fitted the data well in the test set (χ^2^ = 292, *df* = 17; CFI = 0.948; RMSEA = 0.068, SRMR = 0.052), using 3529 cases due to missing data. The path coefficients from perceived intimacy and perceived partner responsiveness to sexual desire are 0.15 and 0.13, respectively. The coefficient from avoidant attachment-related emotional needs to sexual desire is 0.07. The other paths, including the paths representing moderation effects (dashed arrows) are not statistically significant. To check for robustness of the model and for overfitting of the test set, we tested for invariance across test and validation set of respectively the model structure (configural invariance), the equality of the intercepts, and the equality of the regression parameters (van de Schoot et al., 2012). The results of the invariance tests are presented in Table 5.

**TABLE 5:** Testing the invariance of the model across test set and validation set.

**Model constraints**	***Df***	***χ^2^***	***Dχ^2^***	***D(df)***	***Significance***
Configural invariance	34	506			
Intercepts	41	514	8.2	7	0.310
Regression parameters	49	521	7.3	8	0.510

The two sets show configural invariance, and both sets have the same intercepts and the same regression coefficients. The constrained model fitted the data also well in the validation set (χ^2^ = 221, *df* = 17; CFI = 0.952; RMSEA = 0.053, SRMR = 0.042), using 3397 cases due to missing data. The parameter estimates for the regression coefficients are given in Table 6. The explained variance of sexual desire by the predictors in the model was 15%.

**TABLE 6:** Parameter estimates of theoretical model in the validation set.

	***Sexual desire***			
**Predictor variables**	***B***	***SE***	***z***	***p***
Intercept Gender (male) Relationship duration Perceived intimacy Anxious attachment Avoidant attachment Partner responsiveness Intimacy × anxious attachment Intimacy × avoidant attachment *R*^2^ *F*(8,3388)	−0.046 0.597 −0.021 0.146 0.000 0.074 0.133 −0.022 0.019 0.15 77.20	0.024 0.038 0.002 0.024 0.017 0.018 0.024 0.015 0.016	−1.93 −15.61 10.60 6.12 0.00 4.06 5.54 −1.51 1.18	0.054 <0.001 <0.001 <0.001 1.000 <0.001 <0.001 0.130 0.240 <0.001

**FIGURE 3:**
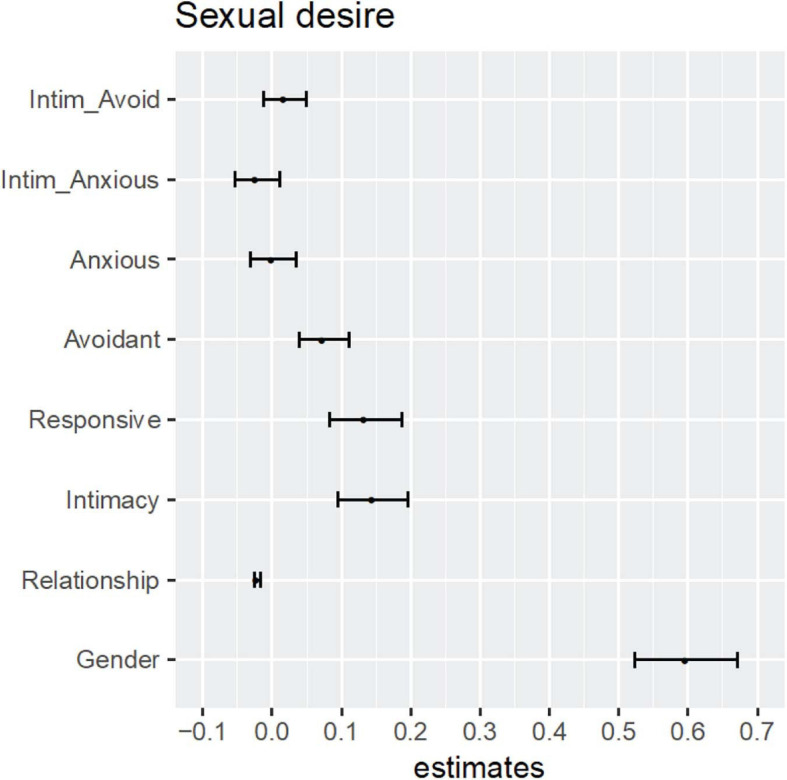
Bootstrap parameter estimates obtained from SEM model on the validation data. Intim_Avoid, Interaction of Perceived Intimacy × Avoidant Attachment; Intim_Anxious, Interaction of Perceived Intimacy × Anxious Attachment; Anxious, Anxious Attachment; Avoidant, Avoidant Attachment; Responsive, Perceived Partner Responsiveness; Intimacy, Perceived Intimacy; Relationship, Relationship Duration; Gender, male effect.

To assess the precision of the regression parameters a bootstrap analysis was run on the validation data to obtain confidence intervals around the parameter estimates. In Figure 3, the results are shown. The strong effect of gender is clearly visible, indicating that men scored higher on sexual desire than women. A small but precise result was seen for relationship duration: longer relations showed slightly lower sexual desire. Higher levels of perceived intimacy and partner responsiveness predicted higher level of sexual desire. The interaction scores of intimacy with anxious and avoidant attachment did not yield significant effects on sexual desire (see Table 6), and did thus not moderate the association between intimacy and sexual desire. However, there was a positive main effect of avoidant attachment-related relational needs on sexual desire.

## Discussion

In this study, we investigated the associations between intimacy, partner responsiveness, and anxious and avoidant attachment on the one hand, and sexual desire on the other hand. Men in our sample reported higher levels of sexual desire than women, also after controlling for age and relationship duration. This effect of gender is in line with previous cross-sectional and experimental research showing higher levels of both proactive or ‘spontaneous’ (Gebauer et al., 2014; Paterson et al., 2014), and responsive (Timmers et al., 2018) sexual desire in men, compared to women. Note, however, that an absence of differences has also been found (e.g., Murray and Milhausen, 2012). Significant effects of sexual orientation and of the interaction of gender and sexual orientation were found on sexual desire. Gender differences in sexual desire across life and relationship stages have been documented in multiple studies (Petersen and Hyde, 2010; McNulty et al., 2019) and cross-sectional data have also shown effects of sexual orientation on sexual desire (Peixoto, 2019). The magnitude of the effects of gender and sexual orientation has, to the best of our knowledge, not been directly compared. Although these effects were too small to be meaningfully interpreted as such, the finding of both a small but significant effect (Cohen, 1988) on sexual arousal of gender and more than ten times smaller effects of sexual orientation and the interaction of gender and sexual orientation is therefore noteworthy. Age and relationship duration were significantly related to sexual desire. In line with earlier research, longer relationship duration predicted lower levels of desire. The association of age and level of sexual desire could not be meaningfully interpreted, due to the extremely small effect size.

The current data supported the hypothesized positive linear associations of perceived intimacy and perceived partner responsiveness with sexual desire. Although perceived intimacy and perceived partner responsiveness were highly correlated, both contributed independently to the prediction of sexual desire. These findings are fully in line with theoretical notions that intimacy and partner responsiveness facilitate the emergence of sexual desire in long-term relationships (Basson, 2000, 2003; Mikulincer et al., 2010; Dewitte, 2012), as well as with previous empirical investigations (Štulhofer et al., 2013; Connaughton et al., 2016). Moreover, the current findings show that both constructs, in addition to showing a strong overlap, may independently contribute to the prediction of sexual desire, lending support to the model of intimacy of Reis and Shaver (1988) that features partner responsiveness as one of the ingredients of a process resulting in the experience of intimacy. Although this model has been investigated in small samples using daily diary methodology (e.g., Laurenceau et al., 1998), it has not been investigated in a large-size sample, such as the present study did.

Anxious attachment-related needs were found to correlate significantly and positively with sexual desire, supporting our hypothesis. This finding seems plausible given that anxiously attached individuals have a strong longing for being close to their significant other. In these persons sexual desire may be predominantly driven by their need for intimacy. Although strong negative feelings inherent in anxiety might dampen sexual desire, this effect may not have shown in our community sample.

An exception was found for non-heterosexual men who did not evidence an association between anxious attachment-related needs and sexual desire. Low statistical power due to the smaller sample size of the latter subgroup is not a very plausible explanation for this finding, as the correlation patterns in non-heterosexual men were otherwise similar to the other subgroups, and significant. This finding is different from those of Zamora et al. (2013) who found significant associations of anxious attachment with passionate and possessive love styles in gay men. Similarly, Passarelli and Vidotto (2016) found a significant positive correlation of anxious attachment and basal sex drive in gay men. A speculative explanation for this observed difference in findings concerns the methods used: in the beforementioned studies attachment was measured using validated questionnaires whereas our measure of attachment was constrained to a small selection of items, that were formulated as attachment-based emotional needs. The wording of the selected items may not have been able to represent the anxious attachment orientations of non-heterosexual men in our sample, while they reflected the attachment orientations of participants in the other subsamples more adequately.

Also supporting our hypothesis, avoidant attachment-related needs correlated negatively with sexual desire, but only in the subsample of heterosexual women. This may, however, be due to the extremely small effect size that only crossed the significance threshold in the largest subsample. These findings are in line with attachment-based models of sexual desire (Mikulincer and Shaver, 2007; Birnbaum, 2010; Dewitte, 2012). Whereas anxiously attached individuals crave for this enhanced intimacy with their partners, avoidantly attached individuals tend to avoid the high level of intimacy that is involved in responding to their partner’s emotional needs.

Attachment orientation was not measured directly in this study. Instead, emotional needs were measured to reflect the psychological dynamics of respectively, anxiously and avoidantly attached individuals. The item wordings “I want my partner to be sensitive to my emotional needs” and “I want my partner to support me emotionally” were considered to reflect central aspects of anxious attachment formulated in terms of emotional needs. However, the items wordings “I am afraid of being emotionally hurt by my partner” and “I want to feel independent of my partner” may be less straightforwardly reflecting avoidant attachment orientation. Nevertheless, exploratory factor analysis showed that both items had high loadings on a single factor, that was distinct from the second factor representing emotional needs related to anxious attachment. Although both items load on the same factor, the first item might also represent anxious attachment-related emotional needs. The need for emotional independence, as expressed in the second item, does more directly represent the avoidant tendency. We speculate that the finding that both items capture the same construct, may be caused by the fear of being emotionally hurt underlying both the need for distance and autonomy in individuals who are avoidantly attached, and the need for proximity in anxiously attached individuals. Anxious and avoidant attachment share the common basis of anxiety and insecurity, and the differences between both attachment orientations may not be absolute. While the underlying anxiety is the same, the coping strategies may be different, with anxiously attached individuals amplifying their anxiety and seeking support, and avoidantly attached individuals attempting to cope with anxiety by creating distance. We consider the construct on which the two items loaded as representing emotional needs related to avoidant attachment due to the clear avoidant nature of the second item (“I want to feel independent of my partner”). Future investigation should clarify if the strong inclination toward emotional autonomy is also caused by fear of being emotionally hurt by one’s romantic partner.

The hypotheses that the link between intimacy and sexual desire would be moderated by attachment-related relational needs were rejected. It can be argued that these effects are much stronger in women and men with clinical levels of anxious and avoidant attachment orientation. A discontinuity between the current community sample and clinical samples might thus speculatively account for the observed null effect.

A strength of the present study is that all associations were found in an online, convenience, community sample of substantial size and heterogeneity, that generated sufficient statistical power to detect even extremely small-sized associations. However, we also acknowledge several limitations of the present study, including its cross-sectional design, that does not allow to investigate fluctuations in both the predictor variables and the criterion variable of sexual desire. To investigate causality in these associations of interest, experimental research is required (Maxwell and Cole, 2007). A second limitation is that the presence of transgender participants cannot be inferred from the way gender was assessed in the demographic questionnaire. This may have resulted in the inclusion of an unknown number of transgender participants in the sample. Another limitation pertains to the way sexual orientation was assessed. By inferring, as we did, sexual orientation from the reply to the question which gender their partner had, bisexual participants and participants with other non-heterosexual orientations could not self-report essential aspects of their sexual orientation. This may have created a bias of unknown size to the sample composition, and subsequently to the conclusions based on it. Still another limitation pertains to the use of unvalidated questionnaires to measures the key constructs, including the measures of attachment-based relational needs that each consisted of only two items. The constructs intended to be assessed may thus not have been captured to their full extent. No data were collected to measure the level of sexual functioning. In combination with the potential self-selection bias (Bogaert, 1996; Plaud et al., 1999) in our sample, as a result of which we may have investigated a relatively well-functioning selection from the adult population, this may have resulted in underestimation of the strength of the observed associations. Responsive sexual desire may be especially important in women who experience sexual problems (cfr., Basson, 2000, 2003), whereas proactive sexual desire may be more representative of individuals without sexual problems, as described by a linear model of sexual functioning (cfr., Masters and Johnson, 1966, 1970). On the other hand, we consider it a strong feature of our study that we were able to validate all observed associations and regression models in an independent data set of sufficient magnitude; all findings were fully replicated.

In sum, several predictions based on the adult attachment model of sexuality were confirmed in an online community sample, including the predictions of positive associations of intimacy, partner responsiveness, and insecure attachment with sexual desire. Moreover, these associations were found to be similar in both genders and independent of sexual orientation. The idea that the relation between intimacy and partner responsiveness with sexual desire would be moderated by attachment orientation could, however, not be confirmed. The cross-sectional design of this study calls for cautious interpretations of the findings and their implications for theory and clinical practice. Future experimental research should be awaited to confirm the causal nature of the observed associations.

## Data Availability Statement

The raw data supporting the conclusions of this article are available at https://osf.io/adgw2/.

## Ethics Statement

This study involved human participants was reviewed and approved by the Ethical Research Board of the Open University of the Netherlands. The participants provided their informed consent to participate in this study.

## Author Contributions

JvL, MD, and SvH contributed to the conceptualization of the study. JvL prepared the data collection. PV performed the data analyses. JvL, MD, PV, and SvH contributed to the writing and reviewing of the manuscript. All authors contributed to the article and approved the submitted version.

## Conflict of Interest

The authors declare that the research was conducted in the absence of any commercial or financial relationships that could be construed as a potential conflict of interest.

## References

[B1] Ågmo A. (1999). Sexual motivation: an inquiry into events determining the occurrence of sexual behavior. *Behav. Brain Res.* 105 129–150. 10.1016/S0166-4328(99)00088-1 10553696

[B2] Arbuckle J. L. (2011). *Amos 20 User’s Guide IBM.* Mount Pleasant, MI: A.D. Corporation.

[B3] Basson R. (2000). The female sexual response: a different model. *J. Sex. Marital Ther.* 26 51–65. 10.1080/009262300278641 10693116

[B4] Basson R. (2003). Biopsychosocial models of women’s sexual response: applications to management of ‘desire disorders’. *Sex. Relationsh. Ther.* 18 107–115. 10.1080/1468199031000061308

[B5] Birnbaum G. E. (2010). Bound to interact: the divergent goals and complex interplay of attsachment and sex within romantic relationships. *J. Soc. Pers. Relationsh.* 27 245–252. 10.1177/0265407509360902

[B6] Birnbaum G. E. Reis H. T. (2012). When does responsiveness pique sexual interest? Attachment and sexual desire in initial acquaintanceships. *Pers. Soc. Psychol. Bull.* 38 946–958. 10.1177/0146167212441028 22517110

[B7] Birnbaum G. E. Cohen O. Wertheimer V. (2007). Is it all about intimacy? Age, menopausal status, and women’s sexuality. *Pers. Relationsh.* 14 167–185. 10.1111/j.1475-6811.2006.00147.x

[B8] Birnbaum G. E. Reis H. T. Mizrahi M. Kanat-Maymon Y. Sass O. Granovski-Milner C. (2016). Intimately connected: the importance of partner responsiveness for experiencing sexual desire. *J. Pers. Soc. Psychol.* 111 530–546. 10.1037/pspi0000069 27399250

[B9] Bogaert A. F. (1996). Volunteer bias in human sexuality research: evidence for both sexuality and personality differences in males. *Arch. Sex. Behav.* 25 125–140. 10.1007/BF02437932 8740519

[B10] Bolger N. Davis A. Rafaeli E. (2003). Diary methods: capturing life as it is lived. *Ann. Rev. Psychol.* 54 579–616. 10.1146/annurev.psych.54.101601.145030 12499517

[B11] Brennan K. A. Shaver P. R. (1995). Dimensions of adult attachment, affect regulation, and romantic relationship functioning. *Pers. Soc. Psychol. Bull.* 21 267–283. 10.1177/0146167295213008

[B12] Brennan K. A. Clark C. L. Shaver P. R. (1998). “Self-report measurement of adult attachment: an integrative overview,” in *Attachment Theory and Close Relationship*, eds Simpson J. A. Rholes W. S. (New York, NY: Guilford Press), 46–76.

[B13] Caprariello P. A. Reis H. T. (2011). Perceived partner responsiveness minimizes defensive reactions to failure. *Soc. Psychol. Pers. Sci.* 2 365–372. 10.1177/1948550610391914

[B14] Clayton A. H. Segraves R. T. Leiblum S. Basson R. Pyke R. Cotton D. (2006). Reliability and validity of the Sexual Interest and Desire Inventory-Female (SIDI-F), a scale designed to measure severity of female hypoactive sexual desire disorder. *J. Sex Mar. Ther.* 32 115–135. 10.1080/00926230500442300 16418104

[B15] Cohen J. (1988). *Statistical Power Analysis for the Behavioral Sciences*, 2nd Edn. Hillsdale, NJ: Lawrence Earlbaum Associates.

[B16] Connaughton C. Mccabe M. Karantzas G. (2016). Conceptualization of the sexual response models in men: are there differences between sexually functional and dysfunctional men? *J. Sex. Med.* 13 453–463. 10.1016/j.jsxm.2015.12.032 26944467

[B17] Conradi H. J. Gerlsma C. Van Duijn M. De Jonge P. (2006). Internal and external validity of the experiences in close relationships questionnaire in an american and two dutch samples. *Eur. J. Psychiatr.* 20 258–269.

[B18] Dewitte M. (2012). Different perspectives on the sex-attachment link: towards an emotion-motivational account. *J. Sex Res.* 49 105–124. 10.1080/00224499.2011.576351 22380584

[B19] Dewitte M. Van Lankveld J. Vandenberghe S. Loeys T. (2015). Sex in its daily relational context. *J. Sex. Med.* 12 2436–2450. 10.1111/jsm.13050 26608879

[B20] Ebesu Hubbard A. S. (2001). Conflict between relationally uncertain romantic partners: the influence of relational responsiveness and empathy. *Comm. Monogr.* 68 400–414. 10.1080/03637750128071

[B21] Feeney J. A. Noller P. (2004). “Attachment and sexuality in close relationships,” in *The Handbook of sexuality in Close Relationships*, eds Harvey J. H. Wenzel A. Sprecher S. (Mahwah, NJ: Lawrence Erlbaum Associates Publishers), 183–201.

[B22] Ferreira L. C. Narciso I. Novo R. F. Pereira C. R. (2014). Predicting couple satisfaction: the role of differentiation of self, sexual desire and intimacy in heterosexual individuals. *Sex. Rel. Ther.* 29 390#x2013;404. 10.1080/14681994.2014.957498

[B23] Gebauer J. E. Baumeister R. F. Sedikides C. Neberich W. (2014). Satisfaction–adaptation principles in sexual desire: exploring gender differences across the life span. *Soc. Psychol. Pers. Sci.* 5 176–184. 10.1177/1948550613490970

[B24] Giles K. R. McCabe M. P. (2009). Conceptualizing women’s sexual function: linear vs. circular models of sexual response. *J. Sex. Med.* 6 2761–2771. 10.1111/j.1743-6109.2009.01425.x 19686428

[B25] Janssen E. Everaerd W. Spiering M. Janssen J. (2000). Automatic processes and the appraisal of sexual stimuli: toward an information processing model of sexual arousal. *J. Sex Res.* 37 8–23. 10.1080/00224490009552016

[B26] Kedde H. (2012). Seksuele disfuncties in Nederland: prevalentie en samenhangende factoren. *Tijdschr. Seksuol.* 36 98–108.

[B27] Kline R. B. (2011). *Principles and Practice of Structural Equation Modeling*, 3rd Edn. New York, NY: Guilford Press.

[B28] La Guardia J. G. Ryan R. M. Couchman C. E. Deci E. L. (2000). Within-person variation in security of attachment: a self-determination theory perspective on attachment, need fulfillment, and well-being. *J. Pers. Soc. Psychol.* 79 367–384. 10.1037/0022-3514.79.3.367 10981840

[B29] Laurenceau J.-P. Barrett L. F. Pietromonaco P. R. (1998). Intimacy as an interpersonal process: the importance of self-disclosure, partner disclosure, and perceived partner responsiveness in interpersonal exchanges. *J. Pers. Soc. Psychol.* 74 1238–1251. 10.1037/0022-3514.74.5.1238 9599440

[B30] Manne S. Ostroff J. Rini C. Fox K. Goldstein L. Grana G. (2004). The interpersonal process model of intimacy: the role of self-disclosure, partner disclosure, and partner responsiveness in interactions between breast cancer patients and their partners. *J. Fam. Psychol.* 18 589–599. 10.1037/0893-3200.18.4.589 15598164

[B31] Mark K. P. Lasslo J. A. (2018). Maintaining sexual desire in long-term relationships: a systematic review and conceptual model. *J. Sex. Res.* 55 563–581. 10.1080/00224499.2018.1437592 29521522

[B32] Masters W. H. Johnson V. E. (1966). *Human Sexual Response.* Oxford: Little, Brown.

[B33] Masters W. H. Johnson V. E. (1970). *Human Sexual Inadequacy.* Boston: Little, Brown.

[B34] Maxwell S. E. Cole D. A. (2007). Bias in cross-sectional analyses of longitudinal mediation. *Psychol. Meth.* 12 23–44. 10.1037/1082-989X.12.1.23 17402810

[B35] McCabe M. P. (1997). Intimacy and quality of life among sexually dysfunctional men and women. *J. Sex Mar. Ther.* 23 276–290. 10.1080/00926239708403932 9427207

[B36] McCabe M. P. Sharlip I. D. Lewis R. Atalla E. Balon R. Fisher A. D. (2016). Incidence and prevalence of sexual dysfunction in women and men: a consensus statement from the Fourth International Consultation on Sexual Medicine 2015. *J. Sex. Med.* 13 144–152. 10.1016/j.jsxm.2015.12.034 26953829

[B37] McNulty J. K. Maxwell J. A. Meltzer A. L. Baumeister R. F. (2019). Sex-differentiated changes in sexual desire predict marital dissatisfaction. *Arch. Sex. Behav.* 48 2473–2489. 10.1007/s10508-019-01471-6 31471791

[B38] Mikulincer M. Shaver P. R. (2007). *Attachment in Adulthood: Structure, Dynamics, and Change.* New York, NY: Guilford Press.

[B39] Mikulincer M. Shaver P. R. Bar-On N. Ein-Dor T. (2010). The pushes and pulls of close relationships: attachment insecurities and relational ambivalence. *J. Pers. Soc. Psychol.* 98 450–468. 10.1037/a0017366 20175624

[B40] Mizrahi M. Hirschberger G. Mikulincer M. Szepsenwol O. Birnbaum G. E. (2016). Reassuring sex: can sexual desire and intimacy reduce relationship-specific attachment insecurities? *Eur. J. Soc. Psychol.* 46 467–480. 10.1002/ejsp.2184

[B41] Muise A. Impett E. A. Desmarais S. (2013). Getting it on versus getting it over with: sexual motivation, desire, and satisfaction in intimate bonds. *Pers. Soc. Psychol. Bull.* 39 1320–1332. 10.1177/0146167213490963 23812928

[B42] Murray S. H. Milhausen R. R. (2012). Sexual desire and relationship duration in young men and women. *J. Sex Mar. Ther.* 38 28–40. 10.1080/0092623X.2011.569637 22268980

[B43] Passarelli M. Vidotto G. (2016). Predictors of interest in sexual and sentimental relationships among gay males: attachment, sex drive, attitudes toward sex and internalized homophobia. *Men Masc.* 19 460–479. 10.1177/1097184X15599661

[B44] Paterson L. Q. P. Jin E. S. Amsel R. Binik Y. M. (2014). Gender similarities and differences in sexual arousal, desire, and orgasmic pleasure in the laboratory. *J. Sex. Res.* 51 801–813. 10.1080/00224499.2013.867922 24588445

[B45] Peixoto M. M. (2019). Sexual satisfaction, solitary, and dyadic sexual desire in men according to sexual orientation. *J. Homosex.* 66 769–779. 10.1080/00918369.2018.1484231 29863980

[B46] Petersen J. L. Hyde J. S. (2010). A meta-analytic review of research on gender differences in sexuality, 1993-2007. *Psychol. Bull.* 136 21–38. 20063924 10.1037/a0017504

[B47] Plaud J. J. Gaither G. A. Hegstad H. J. Rowan L. Devitt M. K. (1999). Volunteer bias in human psychophysiological sexual arousal research: to whom do our research results apply? *J. Sex Res.* 36 171–179. 10.1080/00224499909551982

[B48] R_Core_Team (2016). *R: A Language and Environment for Statistical Computing.* Vienna: R Foundation for Statistical Computing.

[B49] Reis H. T. (2013). “Relationship well-being: the central role of perceived partner responsiveness,” in *Human Bonding: The Science of Affectional Ties*, eds Hazan C. Campa M. I. (New York, NY: Guilford Press), 283–307.

[B50] Reis H. T. Shaver P. (1988). “Intimacy as an interpersonal process,” in *Handbook of Personal Relationships*, ed. Duck S. (Chichester: Wiley), 367–389.

[B51] Reis H. T. Crasta D. Rogge R. D. Maniaci M. R. Carmichael C. L. (2018). “Perceived Partner Responsiveness Scale (PPRS),” in *The Sourcebook of Listening Research: Methodology and Measures*, eds Worthington D. L. Bodie G. D. (New York, NY: John Wiley & Sons, Inc), 516–521.

[B52] Reis H. T. Maniaci M. R. Caprariello P. A. Eastwick P. W. Finkel E. J. (2011). Familiarity does indeed promote attraction in live interaction. *J. Pers. Soc. Psychol.* 101 557–570. 10.1037/a0022885 21381850

[B53] Rosseel Y. (2012). Lavaan: an R package for structural equation modeling. *J. Stat. Softw.* 48 1–36. 10.18637/jss.v048.i02

[B54] Rubin H. Campbell L. (2012). Day-to-day changes in intimacy predict heightened relationship passion, sexual occurrence, and sexual satisfaction: a dyadic diary analysis. *Soc. Psychol. Pers. Sci.* 3 224–231. 10.1177/1948550611416520

[B55] Sand M. Fisher W. A. (2007). Women’s endorsement of models of female sexual response: the nurses’ sexuality study. *J. Sex. Med.* 4 708–719. 10.1111/j.1743-6109.2007.00496.x 17498106

[B56] Segal N. Fraley R. C. (2016). Broadening the investment model: an intensive longitudinal study on attachment and perceived partner responsiveness in commitment dynamics. *J. Soc. Pers. Relationsh.* 33 581–599. 10.1177/0265407515584493

[B57] Shaver P. R. Mikulincer M. (2012). “Adult attachment and sexuality: attachment insecurities bias the functioning of the sexual behavior system,” in *The Wiley-Blackwell Handbook of Couples and Family Relationships*, eds Noller P. Karantzas G. C. (Hoboken, NJ: Wiley-Blackwell), 161–174.

[B58] Shrier L. A. Blood E. A. (2016). Momentary desire for sexual intercourse and momentary emotional intimacy associated with perceived relationship quality and physical intimacy in heterosexual emerging adult couples. *J. Sex Res.* 53 968–978. 10.1080/00224499.2015.1092104 26606678

[B59] Singer B. Toates F. M. (1987). Sexual motivation. *J. Sex. Res.* 23 481–501. 10.1080/00224498709551386

[B60] Spector I. P. Carey M. P. Steinberg L. (1996). The Sexual desire inventory: development, factor structure, and evidence of reliability. *J. Sex. Mar. Ther.* 22 175–190. 10.1080/00926239608414655 8880651

[B61] Sternberg R. J. (1986). A triangular theory of love. *Psychol. Rev.* 93 119–135. 10.1037/0033-295X.93.2.119

[B62] Štulhofer A. Carvalheira A. A. Træen B. (2013). Is responsive sexual desire for partnered sex problematic among men? Insights from a two-country study. *Sex. Relationsh. Ther.* 28 246–258. 10.1080/14681994.2012.756137

[B63] Štulhofer A. Gregurovic M. Pikic A. Galic I. (2005). Sexual problems of urban women in Croatia: prevalence and correlates in a community sample. *Croat. Med. J.* 46 45–51. 15726675

[B64] Tabachnick B. G. Fidell L. S. (2007). *Using Multivariate Statistics*, 5th Edn. Boston, MA: Allyn & Bacon.

[B65] Timmers A. D. Dawson S. J. Chivers M. L. (2018). The effects of gender and relationship context cues on responsive sexual desire in exclusively and predominantly androphilic women and gynephilic men. *J. Sex. Res.* 55 1167–1179. 10.1080/00224499.2018.1456509 29677455

[B66] Toates F. (2014). *How Sexual Desire Works: The Enigmatic Urge.* New York, NY: Cambridge University Press.

[B67] van de Schoot R. Lugtig P. Hox J. (2012). A checklist for testing measurement invariance. *Eur. J. Dev. Psychol.* 9 486–492. 10.1080/17405629.2012.686740

[B68] van Lankveld J. Jacobs N. Thewissen V. Dewitte M. Verboon P. (2018). The associations of intimacy and sexuality in daily life: temporal dynamics and gender effects within romantic relationships. *J. Soc. Pers. Relationsh.* 35 557–576. 10.1177/0265407517743076 29899585 PMC5987853

[B69] Zamora R. Winterowd C. Koch J. Roring S. (2013). The relationship between love styles and romantic attachment styles in gay men. *J. LGBT Issues Couns.* 7 200–217. 10.1080/15538605.2013.812927

